# High Risk HPV Contamination of Endocavity Vaginal Ultrasound Probes: An Underestimated Route of Nosocomial Infection?

**DOI:** 10.1371/journal.pone.0048137

**Published:** 2012-10-24

**Authors:** Jean-sebastien Casalegno, Karine Le Bail Carval, Daniel Eibach, Marie-Laure Valdeyron, Gery Lamblin, Hervé Jacquemoud, Georges Mellier, Bruno Lina, Pascal Gaucherand, Patrice Mathevet, Yahia Mekki

**Affiliations:** 1 Laboratory of Virology, Centre de Biologie et Pathologie Est, Hospices Civils de Lyon, Lyon, France; 2 Gynecology Obstetrics Departement, Hospices Civils de Lyon, Lyon, France; 3 European Public Health Microbiology Training Programme, European Centre for Disease Prevention and Control, Stockholm, Sweden; 4 Preventive Medecine Departement, Hospices Civils de Lyon, Lyon, France; 5 Technical Support Departement, Hospices Civils de Lyon, Lyon, France; IPO, Inst Port Oncology, Portugal

## Abstract

**Background:**

Endocavity ultrasound is seen as a harmless procedure and has become a common gynaecological procedure. However without correct disinfection, it may result in nosocomial transmission of genito-urinary pathogens, such as high-risk Human Papillomavirus (HR-HPV). We aimed to evaluate the currently recommended disinfection procedure for covered endocavity ultrasound probes, which consists of “Low Level Disinfection” (LLD) with “quaternary ammonium compounds” containing wipes.

**Methods:**

From May to October 2011 swabs were taken from endovaginal ultrasound probes at the Gynecology Department of the Lyon University Hospital. During the first phase (May–June 2011) samples were taken after the ultrasound examination and after the LLD procedure. In a second phase (July–October 2011) swab samples were collected just before the probe was used. All samples were tested for the presence of human DNA (as a marker for a possible transmission of infectious pathogens from the genital tract) and HPV DNA with the Genomica DNA microarray (35 different HPV genotypes).

**Results:**

We collected 217 samples before and 200 samples after the ultrasound examination. The PCR was inhibited in two cases. Human DNA was detected in 36 (18%) post-examination samples and 61 (28%) pre-examination samples. After the ultrasound LLD procedure, 6 (3.0%) samples contained HR-HPV types (16, 31, 2×53 and 58). Similarly, HPV was detected in 6 pre-examination samples (2.7%). Amongst these 4 (1.9%) contained HR-HPV (types 53 and 70).

**Conclusion:**

Our study reveals that a considerable number of ultrasound probes are contaminated with human and HR-HPV DNA, despite LLD disinfection and probe cover. In all hospitals, where LLD is performed, the endovaginal ultrasound procedure must therefore be considered a source for nosocomial HR-HPV infections. We recommend the stringent use of high-level disinfectants, such as glutaraldehyde or hydrogen peroxide solutions.

## Introduction

Human papillomavirus (HPV) is now recognized as the major etiological cause of invasive cervical cancer and cervical intraepithelial neoplasia worldwide [1]–[3]. More than 100 human HPV genotypes, classified into high risk (e.g. HPV 16, 18) and low risk types (e.g. HPV 6, 11) [2], are known to infect the anogenital tract [4]. The high risk genotypes have been found to be closely associated with cervical cancer [3], [4] and high grade cervical intraepithelial neoplasia. Worldwide it has been estimated that 70% of cervical cancers are due to HPV types 16 and 18 [1]. Genital HPV infection is mainly a sexual transmitted disease [3], however perinatal transmission from mother to child has been shown as well [5]. So far little is known on a possible nosocomial source of HPV infection.

HPV has the potential for nosocomial transmission due to high resistance and persistence in the environment. It has been shown that HPV retains 30% of its infectivity, even after dehydration for 7 days [6]. More recently, a study has reported that HPV 16 remains infectious for at least 7 days on a wet surface [7]. These results suggest that fomites represent a possible nosocomial source of HPV infection [8]. Indeed the detection of HPV on medical equipment, such as forceps and cryoprobe tips has been reported, despite proper disinfection procedure [9].

Endovaginal cavity ultrasound, seen as a rapid and completely harmless procedure, has become a common medical diagnostic tool. The close contact between the probe and the *cervix uteri* or vaginal wall may represent a potential vector for sexual transmitted infections, such as HPV. In 2010 Kac noticed HPV contamination of endovaginal and endorectal ultrasound probes, despite usage of specific probe covers [10]. French national disinfection guidelines classify covered endovaginal ultrasound probes as non-critical devices and therefore undergo low level disinfection with products such as quaternary ammonium compounds [11]. The use of high level disinfection has been questioned, because of the potential of shortening the life of the transducer, the increased toxicity and a more time intensive disinfection procedure. The objective of our study was to investigate the proportion of contaminated endovaginal ultrasound probes with HPV in order to evaluate the antimicrobial efficacy of the current standard disinfection procedure.

## Materials and Methods

### Study settings

We performed a prospective study, conducted during two periods in one ward of the gynaecology department of the Lyon University Hospital “Femme Mère Enfant”. Permission to enter the wards and collect the samples has been granted by the head-chief of the gyneco-obstetric department. No patient information of any kind has been gathered and no human samples were tested in this study, therefore no patient consent was required by the local ethical committee. During the first period, from the 2^nd^ of May to the 1^st^of July 2011, probe samples were obtained immediately after the endovaginal ultrasound was performed and the probe was disinfected. In the second sampling period, from the 2^nd^ of July to the 10^th^ of October 2011, we assessed a potential risk of HPV transmission to the next patient, with samples being collected just before the probe was used on a new patient. The mean time interval to the previous disinfection procedure was 71 minutes (range: 16 minutes to 8 hours).

### Standard disinfection procedure

The disinfection of the probes was performed by nurses under supervision of the technician in charge of the sampling. Before the study all participating nurses were specifically trained on how to handle the probe in order to avoid contamination via hands or the cover itself. Probes were used with a disposable probe cover (93/42/EEC CE mark) in compliance with national health recommendations [11]. After the examination, the probe cover was carefully removed without contaminating the probe and the probe was disinfected with low level disinfection wipes (Sani-Cloth Active), containing “quaternary ammonium compounds”.

### Sampling

Samples were taken from two endocavity ultrasound probes used on two ultrasound machines (General Electric, VOLUSON-Vaginal Probe RIC5-9D). Probe samples were collected by a specifically trained technician, no more than 15 minutes after the end of the disinfection process. The sample was taken with a dry swab, applied lengthwise across the surface of the probe. The swab was then immediately suspended in a viral transport medium (EMEM) and send to the microbiological lab. Delay between sampling and lab processing did not exceed four hours.

### DNA extraction, amplification and genotyping

The samples were sent to the virology laboratory within less than four hours and stored at −20°C. All samples were extracted using a NucliSens easyMAG instrument (Biomerieux, Marcy L'étoile, France) according to the manufacturer's recommended procedures. HPV amplification and genotyping were conducted using the Genomica SAU (Genomica, Spain) microarray test [12]. This assay allows hybridization of amplified and biotinylated 450 bp fragments from the L1 region of HPV virus. This region is highly conserved and specific for each HPV type [4]. The assay detects 35 HPV types: 20 high risk (HPV 16, 18, 26, 31, 33, 35, 39, 45, 51, 52, 53, 56, 58, 59, 66, 68, 70, 73, 82, 85) and 15 low risk (HPV 6, 11, 40, 42, 43, 44, 54, 61, 62, 71, 72, 81, 83, 84, 89) [11]. Amplification and detection of a human house-keeping gene (cystic fibrosis transmembrane conductance regulator gene) is conducted in each assay in order to ensure sample quality.

Extractions and PCRs for all samples were run in specific series to avoid any risk of contamination from clinical samples. Each series included a negative control in order to test for contamination during the extraction procedure. One swab of each batch, as well as the EMEM medium batch was tested for the absence of HPV contamination.

### Analysis

Statistical analysis was performed with EpiInfo software (V 3.5.1 CDC). Data were expressed as the percentages of positive sample. Inhibited samples were excluded from the calculation.

## Results and Discussion

In our study, no break of the probe covers was detected in any case, and visual inspection did not reveal any presence of blood or body fluids on the probe.

During the first study period from May, 2^nd^ to July 1^st^ 200 probes samples were obtained after endovaginal use and standard disinfection. The PCR was inhibited in two cases. Of the remaining 198 samples, 7 samples (3.5%) were HPV positive. 1 sample showed low risk HPV and 6 samples (3.0%) were positive for at least one high risk HPV type. The detected HPV types include HPV 53, HPV 16, HPV 58 and HPV 31. Two samples revealed more than one HPV type, with a maximum of 4 different HPV types per sample being detected (Table 1). When investigating the sample order, it appeared that three out of four subsequent samples were positive for HPV 58. This observation suggests a persistent probe contamination with the same HR-HPV type, despite the application of three disinfection procedures.

**Table 1:** Results of the 13 Sets of Samples for which HPV was Isolated from Ultrasound Probes after Removal of the Probe Cover under Routine Conditions.

Sample Number	Timing relative to Probe Use	Number of HPV detected	High Risk HPV	Low Risk HPV
1	Before probe use	1	HPV 70	
2	Before probe use	1	HPV 53	
3	Before probe use	1	HPV 53	
4	Before probe use	1		HPV 6
5	Before probe use	1		HPV 6
6	Before probe use	1	HPV 53	
7	After probe use	1		HPV 84
8	After probe use	1	HPV 16	
9	After probe use	1	HPV 53	
10	After probe use	4	HPV 31, HPV 58	HPV 54 HPV 84
11	After probe use	1	HPV 58	
12	After probe use	2	HPV 58	HPV 6
13	After probe use	1	HPV 53	

In the second study period, from July, 2^nd^ to October, 10^th^ 217 samples were collected before the ultrasound examination. One PCR was inhibited. We detected HPV in six out of 216 (2.8%) samples. Four samples were positive with the HR-HPV types HPV 53 and HPV 70 and two samples revealed LR-HPV. We did not detect more than one HPV per sample (Table 1).

The test sample quality control (use for in vitro diagnosis), based on a human housekeeping gene, detected the trace of human DNA in 63/216 (29.2%) and 39/198 (19.7%) samples, before and after the probe use.

This study shows a probe contamination with high risk oncogenic HPV DNA after low level disinfection, despite the use of probe covers. We observed a 2.2% rate of HR-HPV contamination of probes (1.8% before use and 2.5% after use). This result is in agreement with the results observed by Kac G et al [10], who reported 8.2% (95% CI, 4.0%–15.5%) of vaginal-rectal ultrasound probe covers contaminated with HPV, and 0.9% (3/336) contaminated after retrieval of the probe cover, but before low level disinfection (LLD). In a recent publication, Ma ST detects a proportion of 7.5% probes positive for HPV DNA [13]. However this study did not assess the HPV genotype. Our study therefore adds to the evidence, that HR-HPV DNA on endovaginal probes can be routinely detected after low-level disinfection and despite covering of the probe.

The high rate of human DNA on the probes most likely represents human DNA from cells of the genital tract. We tried to avoid any hand contact with the probe during the sampling and the ultrasound examination, but it cannot be ruled out completely. The presence of human DNA from the genital tract on the ultrasound probe can be seen as a marker for a contamination with potential pathogens, attached to cells of the genital tract.

This study may have several limitations. The HPV detection method is based on nucleic acid detection and does not detect the presence of infectious virus particles. The HPV diagnostic faces the same problem, as no HPV cell culture is available up to date. Therefore it is not possible to assess the infectious potential of HPV. Although we are not able to conclude on the infectivity of the detected HPV, considering the potential for HPV resistance on fomites [7] as well as the close and prolonged contact of the probe with the cervix, the detection of HR-HPV DNA on the probe raises concerns.

This risk of swab or probe contamination, due to hand contact was a main concern during the study. Necessary precautions (e.g. training of the sampling staff, use of gloves) were taken to avoid this identified risk, therefore direct HPV contamination of the swab is very unlikely. However, despite the use of gloves, we cannot definitely exclude manual contamination of the probe during probe handling. The main HPV type detected in our study is HPV 53. This high detection rate of HPV 53 is a main feature of the genital HPV genotype distribution of our population in Lyon, as reported in a previous study (12). On the other hand HPV 16, the most prevalent HR-HPV genotype, is detected less than expected. Given the limited number of HPV detected in the study, the HPV genotype distribution should be interpreted cautiously.

The use of dry swabs for sampling could have resulted in a loss of sensitivity. We tried to avoid the problem, by suspending the swab into a viral transport medium (EMEM) and transport the samples into the lab within four hours.

This study was carried out in an almost standard disinfection situation. The staff was aware of the study and this may have strengthened the compliance to the disinfection procedures. Under different circumstances, such as emergency units or wards with a high frequency ultrasound use, compliance may be lower and HPV detection therefore likely to be higher.

The study focused on the detection of HPV on the ultrasound probe. Patients were not sampled in the study, as the main objective was to evaluate the disinfection procedure for covered endocavity ultrasound probes and not routes of transmission.

Presently the French disinfection guidelines [11] allow to classify endovaginal ultrasound probes as non-critical devices, as a probe cover is used and changed for every patient. Non-critical medical equipment is low-level disinfected with substances, such as quaternary ammonium compounds or phenolics. They are not effective against non-enveloped viruses, such as HPV, fungi and bacterial spores [14], [15], [16]. Taken into account the high rate of perforations for commercially available probe covers and to a lesser extent for condoms the “CDC Guideline for Disinfection and Sterilization in Healthcare Facilities” [17] clearly recommends to categorize endovaginal ultrasound probes as semicritical devices. This change in the category implies the use of high-level disinfectants, which also eliminate non-enveloped viruses. Chemical high-level disinfectants, such as hydrogen peroxide, glutaraldehyde or peracetic acid or non-chemical alternatives, such as UVC light can be used [17], [18]. This study was conducted in cooperation with the Hygiene department of the hospital. The results were immediately reported to the responsible authorities. Upon this study it was decided to change the disinfection procedure for endovaginal ultrasound probes from a low-level disinfection to a high-level disinfection procedure. Momentarily a high-level disinfection with UVC light is under evaluation.

In conclusion the study shows a considerable number of endovaginal ultrasound probes being contaminated with HPV after the use of low-level disinfection. Low-level disinfection seems to be inefficient for preventing contamination of endocavity ultrasound probes with HR-HPV and pose a risk of nosocomial transmission of HR-HPV during an ultrasound procedure. We therefore recommend the use of high-level disinfection procedures after each endovaginal ultrasound examinations.
